# Phytochemicals as Immunomodulatory Agents in Melanoma

**DOI:** 10.3390/ijms24032657

**Published:** 2023-01-31

**Authors:** Claudio Tabolacci, Daniela De Vita, Antonio Facchiano, Giuseppina Bozzuto, Simone Beninati, Cristina Maria Failla, Marta Di Martile, Carla Lintas, Carlo Mischiati, Annarita Stringaro, Donatella Del Bufalo, Francesco Facchiano

**Affiliations:** 1 Department of Oncology and Molecular Medicine, Istituto Superiore di Sanità, Viale Regina Elena 299, 00161 Rome, Italy; 2 Department of Environmental Biology, University of Rome La Sapienza, 00185 Rome, Italy; 3 Laboratory of Molecular Oncology, IDI-IRCCS, 00167 Rome, Italy; 4 National Center for Drug Research and Evaluation, Istituto Superiore di Sanità, 00161 Rome, Italy; 5 Department of Biology, University of Rome “Tor Vergata”, 00133 Rome, Italy; 6 Experimental Immunology Laboratory, IDI-IRCCS, 00167 Rome, Italy; 7 Preclinical Models and New Therapeutic Agents Unit, Department of Research and Advanced Technologies, IRCCS Regina Elena National Cancer Institute, 00144 Rome, Italy; 8 Research Unit of Medical Genetics, Department of Medicine, Università Campus Bio-Medico, 00128 Rome, Italy; 9 Operative Research Unit of Medical Genetics, Fondazione Policlinico Universitario Campus Bio-Medico, 00128 Rome, Italy; 10 Department of Neuroscience and Rehabilitation, School of Medicine, University of Ferrara, 44121 Ferrara, Italy

**Keywords:** melanoma, phytochemicals, plant secondary metabolites, immunomodulation, cytokynes, inflammation

## Abstract

Cutaneous melanoma is an immunogenic highly heterogenic tumor characterized by poor outcomes when it is diagnosed late. Therefore, immunotherapy in combination with other anti-proliferative approaches is among the most effective weapons to control its growth and metastatic dissemination. Recently, a large amount of published reports indicate the interest of researchers and clinicians about plant secondary metabolites as potentially useful therapeutic tools due to their lower presence of side effects coupled with their high potency and efficacy. Published evidence was reported in most cases through in vitro studies but also, with a growing body of evidence, through in vivo investigations. Our aim was, therefore, to review the published studies focused on the most interesting phytochemicals whose immunomodulatory activities and/or mechanisms of actions were demonstrated and applied to melanoma models.

## 1. Introduction

Cutaneous melanoma, hereinafter referred as melanoma, is a highly aggressive type of cancer generated from atypical transformation of melanocytes (normal neuroectodermal-derived cells that produce the melanin pigment of skin), often caused by unprotected exposure to ultraviolet (UV) radiation. Melanoma is characterized by high heterogeneity due, for instance, to its mutational status, and poor prognosis due to its capability to rapidly metastasize [1]. Aberrant activation of the BRAF kinase occurs in approximately 50% of melanomas. Among BRAF mutations, the most common consist in single nucleotide mutation resulting in substitution of a valine residue with a glutamic acid in position 600 (BRAFV600E), promoting melanomagenesis through the constitutive activation of ERK and MEK signaling pathways [2]. Several molecularly targeted BRAF inhibitors (BRAFi), including Vemurafenib, Dabrafenib, and Encorafenib, have efficacy in treatment of metastatic melanoma. Targeted therapy with MEK inhibitors (MEKi), including Trametinib, Binimetinib, and Cobimetinib, in combination with BRAFi results in an increase of median progression-free survival. However, resistance to these agents invariably develops [2].

Melanoma is an immunogenic tumor that, during its progression, overexpresses several melanoma-associated antigens (e.g., MAGE, S100, tyrosinase, Melan-A/MART-1, gp100, CDK4) recognized by lymphocytes [3], and for this reason it shows, among other tumors, one of the highest sensitivity to immunotherapies [4]. Unfortunately, despite melanoma’s immunogenicity, this tumor develops several immune escape mechanisms, including a tight molecular crosstalk with cells of the immune system due to autocrine and paracrine growth factors and cytokines secreted by melanoma and stromal cells [5].

**Figure 1:**
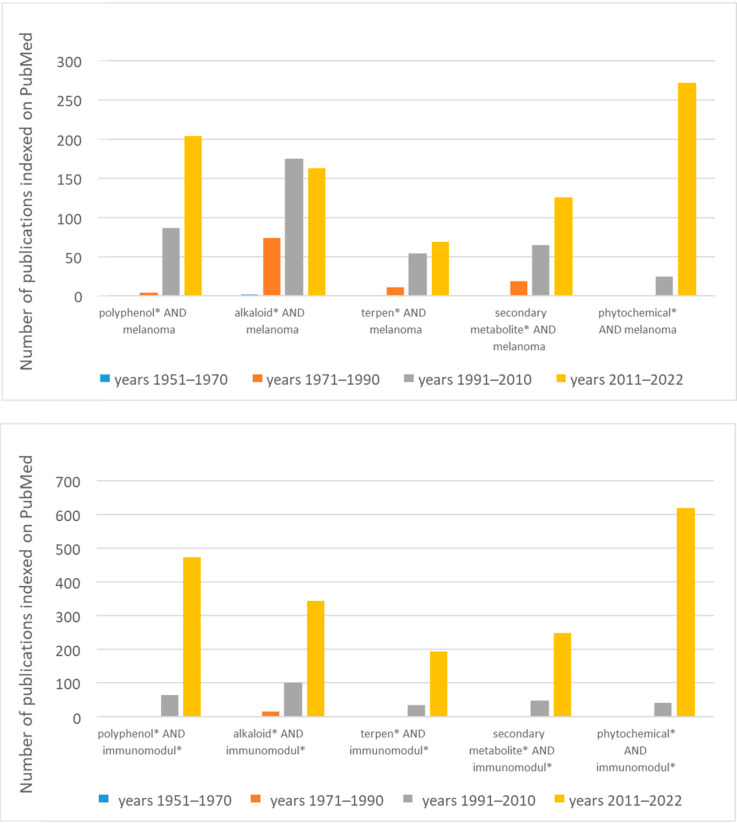
Number of publications indexed on PubMed is reported on four groups: those published between 1951–1970, 1971–1990, 1991–2010 or from 2011 up today (2011–2022: please note that the last one refers to only 11 years), filtered by containing within title/text the name of a category of natural compounds. In upper panel the second search criterion was “melanoma” while in the lower panel the second criterion was “immunomodul*”.

Interestingly, several studies support the immunomodulation activity of plant secondary metabolites underlining their effects on different population of immune cells [6,7,8,9]. The interest of the scientific community in phytochemicals has been rapidly growing in the last years, as shown in Figure 1 where the number of published studies indexed on PubMed filtered for “melanoma” or “immumodulation” keywords is reported.

Interestingly, the alkaloids were, among the phytochemicals, the first category of natural compounds whose activities were studied for application on melanoma, as indicated by the number of published studies before 1990. On the other hand, the studies regarding the immunomodulatory activities of all the phytochemicals (mainly the polyphenols) show a marked increase within the last years (see the bars related to 2011–2022, which interestingly refer to only 11 years), indicating the large and growing interest of scientific community in this specific field of investigation.

This review discussed the recent advances in melanoma immunotherapy and inflammation, highlighting mainly the immunomodulatory effects of natural compounds on melanoma cells and their mechanisms of action. Moreover, this review focused on plant extracts and secondary metabolites with modulating effects on cytokines/chemokines secretion, their application as anti-inflammatory drugs, and their immune-sensitizing effects in melanoma models.

## 2. Immunomodulation and Immunotherapy of Melanoma

### 2.1. Melanoma and Interaction with Tumor Microenvironment

**Figure 2:**
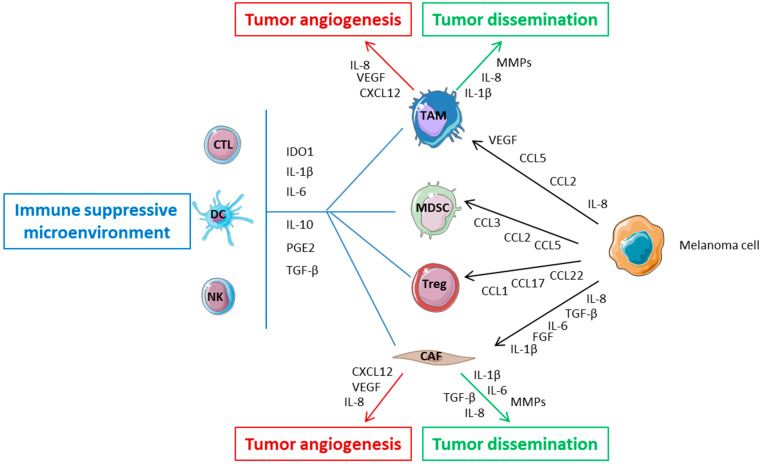
Melanoma cells modulate the communications with cells of the tumor microenvironment through the release of soluble factors in order to promote tumor dissemination, angiogenesis and the establishment of an immune suppressive niche. Cytotoxic T lymphocyte (CTL), dendritic cell (DC), natural killer cell (NK), tumor-associated macrophage (TAM), myeloid-derived suppressor cell (MDSC), T regulatory cell (Treg), cancer-associated fibroblast (CAF), interleukin 8 (IL-8), 1β (IL- 1β), 6 (IL-6), 10 (IL-10), prostaglandin E2 (PGE2), C-X-C motif chemokine 12 (CXCL12), vascular endothelial growth factor (VEGF), tumor growth factor β (TGF- β), metalloproteinases (MMPs), fibroblast growth factor (FGF) and chemokine C-C ligand 1 (CCL1), 2 (CCL2), 5 (CCL5) and 17 (CCL17). Parts of the figure are drawn using pictures from Servier Medical Art (https://smart.servier.com, (accessed on 18 December 2022).

The capability of melanoma to grow, disseminate, develop drug resistance, and attenuate immunosurveillance is strictly dependent on the heterogeneity of the tumor tissue, composed of both tumor cells and the surrounding microenvironment [10]. Tumor microenvironment (TME) can be considered as a complex and dynamic system, composed of non-cancerous stromal (fibroblasts, endothelial and lymphatic cells) and immune (T lymphocytes, B lymphocytes, myeloid-derived suppressor cells (MDSCs), dendritic cells, natural killer, macrophages, neutrophils) cells, extracellular matrix proteins, growth factors and nutrients. Melanoma cells and those of the TME communicate bi-directionally thanks to cell–cell contacts or to the release of soluble factors or exosomes, dictating the fate of melanoma through the regulation of tumor growth, metastatization, angiogenesis and the establishment of an immunosuppressive microenvironment. These functions are mainly carried out by a plethora of soluble factors mediating autocrine and paracrine communications (Figure 2) and, among them, interleukin (IL)-1β, IL-8, IL-6, vascular-endothelial growth factor (VEGF), C-C Motif Chemokine Ligand 2 (CCL2; or monocyte chemoattractant protein-1, MCP-1), CCL5 (RANTES) and tumor necrosis factor (TNF)-α play a pivotal role.

The establishment of an immunosuppressive microenvironment is also mediated by the upregulation of the inhibitory immune checkpoints (PD-L1/PD-1, CTLA-4, TIGIT, LAG-3) by melanoma cells or those of the surrounding microenvironment [11]. Currently, the relevance of NF-kB, JAK/STAT, and HIF pathways in the regulation of almost all of the above-mentioned mediators is now well established [12,13,14]. Taking advantage of these biological characteristics and the genetic background, mostly represented by BRAF mutations in about 50% of melanoma patients, immunotherapy and targeted therapy have been developed as standard therapies for melanoma patients with advanced disease. In particular, immunotherapy, a therapeutic approach boosting or suppressing the immune responses, represents a valid approach to treat melanoma through the inhibition of immune checkpoint inhibitors (ICI) or the modulation of cytokines/cytokine receptors axes [15].

Melanoma, as previously mentioned, is a skin cancer derived from neoplastic transformation of melanocytes, the cells specialized to synthetize and to package the melanin and to transfer it to dermal keratinocytes [16]. It is noteworthy that in mammalian melanocytes, two types of melanin with different behaviors are present, namely eumelanin and pheomelanin [17]. The quality and the amount of melanins confer the individual protection to UV radiation and their role in melanoma progression has been recently reviewed [18,19]. The main role of melanin consists of photoprotection by filtering UV sun radiation [18] but it was demonstrated that the melanin’s role in skin is more complex. In fact, beside the UV filtering and reactive oxygen species (ROS) scavenging actions, other interesting functions have been demonstrated, since, for instance, it was shown that the presence of melanin was able to lower melanoma cell invasiveness by reducing cell elasticity and increasing cell stiffness [20]. On the other hand, the melanin modified by the UVB radiations may induce DNA damage in melanoma cells, thus promoting their immortalization and the carcinogenetic process, supporting the knowledge that melanins’ balance and functions may play a double-faced role for melanoma pathogenesis, progression and metastatic dissemination [21]. Therefore, targeting melanin is an anti-melanoma strategy recently reviewed [22], in a complex scenario recognizing a dual role of melanin, both as a protective molecule against UV-induced damages, on the one hand, and as a necessary molecule for the malignant transformation of melanocytes, on the other hand [19,23]. Moreover, in a melanoma setting the control of the immune response may be achieved via the control of melanin synthesis. Therefore, melanogenesis represents an interesting target since it is directly connected with innate immunity via Toll-Like receptors [24]. Several studies investigated how the immune system is related to melanogenesis in a melanoma setup [25] or addressed melanin synthesis in lymphoid organs in salmon [26] and in insects [27], further supporting the link between melanogenesis and the immune system. On the other hand, it is remarkable that melanin, as well as some key factors of the melanogenesis cascade, may play an important role in the mechanisms of immunosuppression by acting directly on the immune cells or through the inhibition of secretion of inflammatory cytokines [21,28,29].

### 2.2. Immune Checkpoints

**Figure 3:**
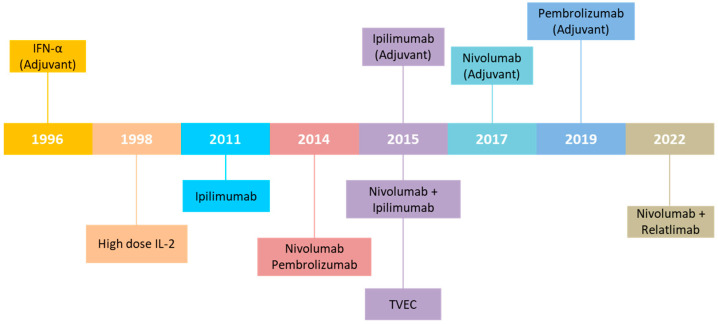
Timeline indicating immunotherapies approved by the FDA for the treatment of patients affected by melanoma (https://www.accessdata.fda.gov/scripts/cder/daf/index.cfm, (accessed on 20 December 2022).

The year 2011, with the approval of the first ICI, marked a revolution in the treatment of patients with metastatic melanoma. The demonstration that tumors are able to activate the normal pathways that regulate T-cell inhibition, the so-called immunological checkpoints, has provided new targets for immunotherapy [30] (Figure 3).

In this context, two immunological checkpoints become the main therapeutic targets for melanoma: cytotoxic T-lymphocyte antigen 4 (CTLA-4) and programmed cell death protein-1 (PD-1). Ipilimumab, a monoclonal antibody directed against CTLA-4, a negative regulator of early phases of T lymphocyte activation in lymph nodes, was approved by the FDA in 2011 for the treatment of patients with advanced melanoma [31]. In 2014, FDA approved Nivolumab and Pembrolizumab, two monoclonal antibodies directed against PD-1, a protein expressed on the surface of T lymphocytes that, through the binding with its two ligands PD-L1 and PD-L2, is able to suppress the activation of T lymphocytes in the peripheral tissues [30]. These ICI have been shown to be superior in terms of efficacy and tolerability compared with Ipilimumab. In terms of survival, Nivolumab and Pembrolizumab resulted in an Overall Survival (OS) rate of 70–75% and approximately 40% after 1 and 5 years, respectively [32,33]. Evidence that activation of the PD-1 and CTLA4 pathways occurs at different stages of T lymphocyte maturation paved the way for combining Ipilimumab and Nivolumab, approved by the FDA in 2015 for unresectable or metastatic melanoma patients, based on the results of the phase III CheckMate 067 trial. The long-term survival analyses demonstrated that 6.5-year OS rates were 50%, 43% and 23% in the combination arm, compared with Nivolumab or Ipilimumab groups, respectively [34,35,36].

Another immunological checkpoint that is gaining therapeutic importance is lymphocyte-activation gene 3 (LAG-3). Recently, the FDA approved the use of Relatlimab, an anti-LAG-3 monoclonal antibody, in combination with Nivolumab for the first-line treatment of advanced melanoma. This approval was triggered by the results of the phase II/III RELATIVITY 047 clinical study, in which the combination of Relatlimab + Nivolumab showed an advantage in terms of median progression-free survival (PFS) compared with single treatment with anti-PD1 (6.7 months in the combination arm vs. 3 months in the Nivolumab one) [37]. The expression of nine immune checkpoints and their relation to overall survival has been recently investigated in 31 cancer types, incuding melanoma [38].

Nowadays, several clinical trials are ongoing to test the efficacy of the combinations between ICI and other agents. Notheworthy was the combination of ICI and targeted therapies in patients carrying BRAF V600 mutation. In this context, randomized phase II SECOMBIT (https://www.clinicaltrials.gov/ct2/show/NCT02631447, (accessed on 15 December 2022), phase III DREAMseq (https://clinicaltrials.gov/ct2/show/NCT02224781, (accessed on 15 December 2022), phase II EBIN (https://www.clinicaltrials.gov/ct2/show/NCT03235245, (accessed on 15 December 2022) and phase II ImmunoCobiVem (https://clinicaltrials.gov/ct2/show/NCT02902029, (accessed on 15 December 2022) trials have been investigating the most effective sequence of administration of ICI and targeted therapies.

Regarding the adjuvant setting, it was expected for cases at high risk of recurrence (stage IIB, IIC) and in the case of positive metastatic lymph nodes (stage III) and for resected stage IV [39]. Treatment with low doses of interferon α (IFN-α) is now used as adjuvant therapy only in cases of ulcerated or stage IIB and IIC melanoma, for which new-generation adjuvant therapies (target therapy and/or immunotherapy) are not approved. Starting from 2015, ICI were also approved by the FDA for adjuvant therapy in melanoma.

Another immunotherapy approach consists of vaccines. In particular, in 2015 talimogene laherparepvec (T-VEC) was approved by the FDA for the treatment of unresectable lesions in patients with recurrence. This approval was based on the results of phase III OPTIM trial demonstrating an Overall Response Rate (ORR) rate of 26% in T-VEC treated patients compared with 5.7% in granulocyte-macrophage colony-stimulation factor (GM-CSF) treated patients [40]. Long-term survival curves showed an OS rate at 5 years of 33% [41].

### 2.3. Cytokines

Cytokines comprise a large and heterogeneous group of molecules produced by immune, stromal and tumor cells and, consequently, are mainly involved in the communication of tumor cells with the TME. This group of molecules includes chemokines, TNFs, IFNs, interleukins, and growth factors. The genetic and molecular background of melanoma cells have been reported to affect the expression and activity of several cytokines. This is the case of BRAF mutations in melanoma cells correlating with enhanced levels of immunosuppressive mediators such as IL-6 and IL-10 [42] or anti-apoptotic Bcl-2 and Bcl-xL proteins, regulating the expression of several interleukins, such as IL-8 and IL-1β [43,44]. Pro-tumor cytokines are also abundant in the secretome of drug-resistant melanoma cells [45]. Several cytokines, including monocyte chemoattractant protein 1 (MCP-1), TNF-α [46], IL-6 [47] and IL-2 [48] have been reported to be predictive biomarkers for the response of melanoma patients to ICI treatment or for melanoma metastases [49].

Before the advent of ICI, immunotherapy in the treatment of advanced melanoma consisted of the use of high-dose IL-2 and adoptive cell therapy with autologous tumor-infiltration lymphocytes, with ORR of about 15% and up to 50%, respectively [50,51,52,53]. Nevertheless, only a subset of patients treated with these approaches reached long-term responses [54]. At present several clinical trials using cytokines or their antagonists are ongoing in order to identify new therapeutic approaches. Among the cytokines or cytokine/receptor pathways used in clinical trials for the treatment of metastatic melanoma we must mention IL-2, IL-8, IL-10, IL-12, IFN-α and TNF-α, as well as tumor growth factors. In particular, after the first FDA-approved clinical trial using IL-2 [55], different trials were conducted in order to take advantage of the immunomodulatory properties of specific cytokines. The PEGlylated form of IL-2, Bempegaldesleukin, showing less toxicity and longer half-life than IL-2, was well tolerated and induced activation of the immune response [56] and led to the approval of clinical trials in which it is combined with anti-PD-1/PD-L1/CTLA-4 agents. Very recently, phase II/III studies demonstrated the clinical activity of Bempegaldesleukin plus Nivolumab as a first-line therapy in advanced melanoma [57,58]. Several other clinical trials using Bempegaldesleukin in combination with ICI are ongoing for melanoma treatment (https://clinicaltrials.gov/ct2/show/NCT03635983, (accessed on 15 December 2022); https://clinicaltrials.gov/ct2/show/NCT03435640, (accessed on 15 December 2022). As cytokines play a relevant role in the expansion and survival of lymphocytes, both high-dose and low-dose IL-2 have been used for adoptive cell therapy using autologous tumor-infiltrating lymphocytes for melanoma treatment: a systematic review and meta-analysis evidenced the ability of high dose-IL-2 to induce durable clinical benefit when combined with adoptive cell therapy [54].

In addition, PEGylated IL-10 has been found to induce systemic immune activation in melanoma patients, and its use in combination with Nivolumab induced a manageable toxicity and preliminary anti-tumor activity in melanoma patients (10% patients with objective responses) [59]. Another cytokine reported to activate the immune system through CD8^+^ memory T cell infiltration of melanoma lesions is represented by IL-12 [60]. Other interleukins, such as IL-7 [61,62], IL-15 [63,64] and IL-21 [65]) and GM-CSF [66] have been tested in clinical trials with no evident signal of efficacy. Interestingly, IL-7 and IL-15 have been reported to show higher efficacy when used in adoptive cell therapy and compared with IL-2 [67]. As concerns IFN-α, in 1996 it was the first therapy approved by FDA for the treatment of advanced melanoma [68]. Then, less toxic and more efficacious PEGylated IFN-α was approved by the FDA [69]. Regarding the clinical benefit of IFN-α in the adjuvant therapy of high-risk resected melanoma patients, this is a subject of debate: several results indicate improved overall and recurrence-free survival [70], while others report low or no effect on overall survival [71]. This indicates the need of further study to identify predictive factors to be used for the selection of patients who probably benefit from adjuvant IFN-α therapy. In recent trials, IFN-α was combined with ICI: no clinical efficacy for resected melanoma was observed when an adjuvant treatment of IFN-α was compared to Pembrolizumab [72], while promising evidence of clinical benefit was reported when PEG-IFN was combined with Pembrolizumab in PD-1-naïve metastatic melanoma [73]. Several trials are ongoing to further investigate ICI/PEGylated IFN-α combination in advanced melanoma.

Additionally, results from clinical trials aimed to block cytokines or their receptors could warrant further studies: this is the case of three phase I trials targeting: (i) IL-8 [74], (ii) TGF-β [75] and (iii) TNF-α isoforms in combination with nivolumab and ipilimumab [76], showing both biological activity and clinical efficacy.

## 3. Classification and Extraction Methods of Plant Secondary Metabolites

Secondary metabolites are grouped into various classes according to their chemical structure and biosynthetic pathway. The main groups are alkaloids, phenolic compounds, terpenes and terpenoids. Alkaloids are organic compounds that have, with few exceptions, a basic character containing at least one nitrogen atom, preferably in a heterocycle [77]. These chemical properties confer weak alkaline behaviors to most alkaloids, although in some cases it is possible that they behave neutral or weakly acidic characteristics. In any case, due to the complexity of the chemical structure, a precise and strictly defined chemical characterization is not easy, since many phytochemicals might be correctly classified within one or another class, or, in several cases, on the edge between two adjacent groups (see for instance chromones and flavo-alkaloids) [78]. These summarized chemical features ensure a moderate solubility in water. Even if a complete classification of all known alkaloids is difficult to achieve, a classification system is based on the family of plants where they are present: alkaloids are widespread in angiosperm dicots, including Papaveraceae, Solanaceae and Apocynaceae, and to a lesser extent in monocots [79]. According to a biogenesis-based classification, they can be divided into (i) true alkaloids bearing a nitrogen ring and deriving from amino acids, (ii) protoalkaloids, derived from amino acids, whose nitrogen is not part of a ring, and ( iii) pseudoalkaloids whose skeleton does not derive from amino acids. From a chemical point of view, alkaloids can be divided in classes based on the nitrogen heterocycle, such as indole, xanthine, tropane and isoquinoline, just to mention a few [80]. Among the different categories of secondary metabolites produced by plants, alkaloids represent one of the richest sources of bioactive molecules [81]. Moreover, many synthetic alkaloids derivatives (e.g., vinpocetine) are also available, whose current use for research and/or clinical purposed is often justified by their lower production costs compared with compounds extracted from plants [82].

**Figure 4:**
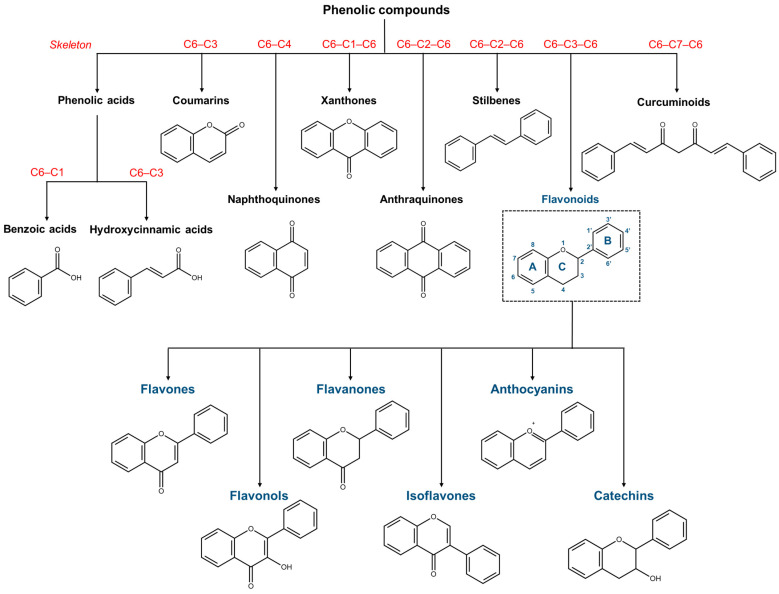
Classification and chemical structure of phenolic compounds. Basic skeleton structure of flavonoids and their principal classes are shown.

Phenolic compounds represent a class of ubiquitously distributed metabolites with a huge molecular variability, bearing one or more hydroxyl groups linked to a benzene ring. They can be divided into groups (Figure 4) according to their biosynthetic precursors: acetyl-CoA and shikimic acid. However, the common classification is based on the number of the carbon in the skeleton, as proposed by Harborne and Simmonds [83].

Therefore, phenolic compounds include phenolic acids, coumarins, lignans, anthraquinones, flavonoids, stilbenes and tannins. Phenolic acids include hydroxybenzoic acid with a C6–C1 structure, such as salicylic acid, and hydroxycinnamic acid (C6–C3), e.g., caffeic and ferulic acid [84]. Coumarins are benzopyrones (1,2-benzopyrones or p-c-1-benzopyran-2-ones) that, having a skeleton C6–C3 [85], are classified as phenylpropanoids, together with hydroxycinnamic acids. The dimerization C6–C3 units lead to lignans, neo-lignans and nor-lignans [86]. Flavonoids are polyphenols with a C6–C3–C6 skeleton and flavane nucleus, consisting of two benzene rings (named A and B) linked through a pyran ring (C) [87]. The most common subclasses of flavonoids are anthocyanins, flavones, flavonols, flavanones, isoflavones and flavanols [84]. Flavonoids exist in plants in the form of aglycones or glycosides (mostly *O*-glycoside or *C*-glycoside forms). In particular, the presence of the sugar residue influences their bioavailability and metabolism, improving their water solubility and their absorption from the gastrointestinal tract [88]. Having a C6–C3–C6 skeleton, chalcones also belong to the flavonoids family and consist of two aromatic rings linked by a three-carbon α,β-unsaturated carbonyl system. Stilbenes, such as the well-known resveratrol, are phenolic compounds with a C6–C2–C6 skeleton where two benzene rings are joined by an ethylene moiety, usually as *trans* isomers [84,89]. Anthraquinones (C6–C2–C6) are anthracene derivatives bearing at positions 9 and 10 two keto groups, while the reduction at position 10 gives anthrones [90]. The replacement of C-10 with one oxygen gives xanthones with a C6–C1–C6 carbon skeletal structure where two aromatic rings fuse together through an oxygen and a carbonyl group [91]. A simpler polycyclic system, having a C6–C4 skeleton, is represented by naphthoquinones that most commonly occur as para isomers, 1,4-naphthoquinones [92]. Tannins are a heterogeneous group of polyphenolic compounds with high molecular weight, known as condensed tannins, including oligo- and poly- flavonoids, and hydrolysable tannins, consisting of esters of gallic acid and ellagic acid glycosides [93]. Finally, plant polyphenols include curcuminoids, a class of diarylheptanoids (C6–C7–C6) isolated from *Curcuma longa* L., whose curcumin is the major component [94].

Terpenes are a large class of secondary metabolites with a chemical structure based on repetition of C5 units, isopentenyl diphosphate (IPP) or dimethylallyl diphosphate (DMAPP) [95]. Unlike terpenoids, which can include heteroatoms such as oxygen and a different structural rearrangement, terpenes are hydrocarbons. However, it is common to use the term “terpene” to also include terpenoids. Structurally, terpenes can be found as acyclic or cyclic molecules [96]. Depending on the number of C5 units, terpenes can be classified as monoterpenes (2 × C5 units), sesquiterpenes (3 × C5 units), diterpenes (4 × C5 units) or triterpenes (6 × C5 units) [97]. Terpenes with lower molecular weight (monoterpenes and sesquiterpenes) are volatile and, together with other volatile metabolites such as alcohols, aldehydes, ketones and phenols constitute the essential oil [98].

Other secondary metabolites are produced by plants even if with a limited distribution in the kingdom. Among them, glucosinolates are a group of sulfur- and nitrogen-containing compounds bearing a β-D-thioglucose group, a sulfonated oxime and an aliphatic or aromatic aglycone side chain derived from amino acids, usually methionine, tryptophan or phenylalanine. Glucosinolates are found in the Brassicaceae family where they act as defence molecules: indeed, damaged tissues release a β-thioglucosidase named myrosinase that hydrolyzes glucosinolates to hydrolysis products, e.g., isothiocyanates with a characteristic pungent flavor [99]. Thiosulfinates are a group of sulfur-containing compounds, typically found in Allium spp. In particular, allicin is a thiosulfinate responsible for the odor of garlic (*Allium sativum* L.). In damaged tissues, it is produced from an odourless amino acid (alliin) by a reaction catalyzed by alliinase [100].

**Figure 5:**
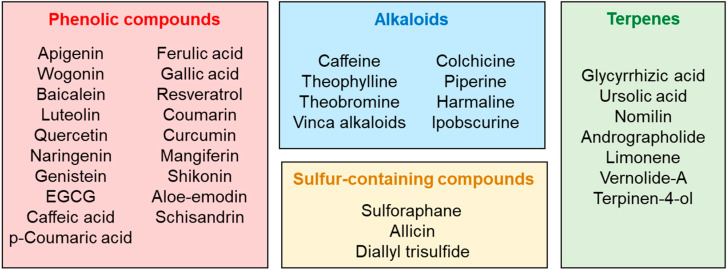
Main secondary metabolites with immunomodulatory activity on melanoma addressed in this paper.

The principal extraction methods of mentioned phytochemicals are listed in Table 1. The main secondary metabolites, with immunomodulatory properties on melanoma, cited in this review are listed in Figure 5.

## 4. Alkaloids

Among the several natural compounds behaving as immunomodulators, alkaloids are some of the most powerful, as reported in studies with in vitro or in vivo cancer models [112,113]. In this section, the most important groups of alkaloids showing immunomodulatory effects are reported.

### 4.1. Methylxanthines

**Figure 6:**
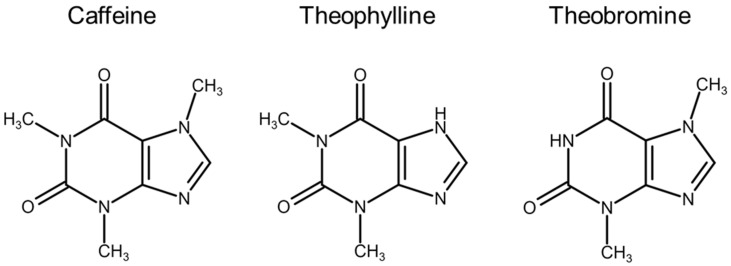
Chemical structure of methylxanthines: caffeine (1,3,7-trimethyl-), theophylline (1,3-dimethyl-) and theobromine (3,7-dimethyl-)xanthine.

Methylxanthines (MXs) are organic molecules whose structure can be traced back to that of a purine base, xanthine, to which one or more methyl groups are linked (Figure 6).

The number and position of these substituents, on the heterocyclic compound, help to distinguish one from the others. Caffeine, theophylline and theobromine are, in fact, three closely related plant alkaloids, and present in nature in coffee plants (*Coffea Arabica* L.), and other species similar to it, tea (*Camellia sinensis* [L.] Kuntze) and cocoa (*Theobroma cacao* L.), whose seeds and fruits are typically obtained and isolated. The chemical structure of these MXs makes them suitable for interaction with numerous and specific biological targets, present in various districts of our body, as well as being chemically very interesting as a starting point for the development of new derivatives in the pharmaceutical field. Their action is mainly determined at the level of the central nervous, cardiovascular and endocrine systems, with an increase in wakefulness and attention, stimulation of the bulbar respiratory centers and release of neurotransmitters [114].

MXs are well known for their psychostimulant properties [115]. Although the immunomodulatory properties of many of the MXs have been extensively described, very few studies have investigated the effects of caffeine on immune function. Caffeine has recently been reported to suppress some of the typical functions of human lymphocytes and macrophages in vitro [116]. Numerous rodent studies have highlighted the effects of theophylline on various immune parameters, such as antibody production, hypersensitivity, natural killer cell cytotoxicity and TNF-α production [117]. Furthermore, there is also much evidence demonstrating that some MXs induce differentiation and apoptosis in some tumor cell lines, but the molecular mechanisms for these actions have not yet been well investigated. In this regard, the interest aroused by MXs as anti-neoplastic agents is growing. MXs currently represent one of the elements capable of interfering with the growth of various cancer cell lines, including melanoma. Indeed, one of the known effects of MXs on tumor cells in vitro is a disturbance of the normal proliferative activities of the treated cells [118]. Moreover, MXs enhanced the activity of tissue transglutaminase (TG2), a ubiquitous enzyme involved in several stages of carcinogenesis [119], reducing the metastatic potential of melanoma cells [120].

The effect of theophylline treatment on the pigmentation characteristics and growth of two B16 melanoma cell lines, HFH-18 and P/140, confirmed that this molecule inhibits cell growth [121]. The results of these experiments demonstrated that in response to theophylline treatment there was an increase in tyrosinase synthesis with induction of cell differentiation. In the mouse model, implanted with B16-F10 melanoma, it was observed that the anti-neoplastic activity of theophylline essentially takes place by preventing the neovascularization of the tumor. Furthermore, this study also highlighted that theophylline did not show any direct toxicity to the host, significantly reducing the volume of the tumor mass. The histological analysis highlighted the blocking of endothelial cell proliferation, thus preventing tumor neovascularization [122]. The anti-tumor activity of MXs was studied through an additional approach [123] to evaluate how the tumor cell is affected in the host by the presence of these compounds. To this end, computerized image analysis was performed on histological sections of mouse lungs (C57BL6/N) that had been injected intravenously (i.v.) with B16-F10 melanoma cells. It was possible to evaluate the growth inhibition rate and the anti-invasive ability of theophylline, caffeine and theobromine, thus obtaining more accurate data than those obtained from the common macroscopic count of superficial metastatic foci. The anti-tumor activity of the MXs examined was evaluated considering two morphometric parameters: the “growth index”, related to the size of the metastases and the “invasion index” related to the frequency of the foci. Based on this evaluation, it was possible to highlight an evident relationship between the morphometric parameters and two markers of tumor invasion and cellular differentiation, hydroxyproline and TG2, respectively, showing that the anti-melanoma effects of MXs were not only on the cancer cells, but also on their interaction with the surrounding microenvironment [123]. Interestingly, more recently, TG2 has been demonstrated to mediate UV-induced inflammation by increasing inflammatory cytokines (such as TNF-α, IL-6 and IL-8) production in the skin [124].

The immunomodulatory MXs effects have also been confirmed by more recent studies involving human melanoma initiating cells (MICs), showing caffeine’s ability to induce a significant reduction of MIP-1α, MIP-1β, IL-1β, IP-10 and RANTES secretion by MICs within the conditioned media [125]. On the other hand, another recent study on human A375 and SK-MEL-30 cells, on their derived spheroids and on patient-derived MICs confirmed the role of tissue transglutaminase activity as an important differentiation marker and a significant theophylline immunomodulatory action by interfering in the cross-talk between TME and MICs [126]. Interestingly, in this study a significant melanogenetic effect of theophylline on MICs was reported.

It is noteworthy that MXs have been also the natural compounds from which several derivatives with potent anti-neoplastic and immunomodulatory activities have been synthetized. This is the case of melanogenesis-stimulating hormone 3-isobutyl 1-methylxanthine (IBMX) [127] as well as of Pentoxifylline (PTX) [128].

### 4.2. Other Alkaloids

Vinca alkaloids (VAs) are a group of organic compounds isolated from the Madagascar periwinkle plant (*Catharanthus roseus* G. Don (*Vinca rosea* L.)) and are either natural or semisynthetic compounds. They were used for many years and are still used in anticancer therapy [129] including melanoma as combined agents in chemotherapy [130]. These molecules at high concentrations are microtubule-destabilizing agents. For some of them (vincristine, vindesine, and vinorelbine) the molecular mechanism of acquired resistance in VAs treated melanoma cells was reported [131]. A potent anti-inflammatory activity of some VAs (e.g., for vincamine and its synthetic derivative vinpocetine [132]) has been reported, as well as a direct action on melanoma cells’ viability [133]. Moreover, its anti-inflammatory activity through the inhibition of the NF-kB pathway was also reported in acute ischemic stroke patients [134].

Colchicine is a water-soluble alkaloid derived from Autumn Crocus (*Colchicum autumnale* L.) and from Glory Lilly (*Gloriosa superba* L.) plants, largely used in the past as an anti-inflammatory agent and recently suggested as a potential anti-mitotic agent for anti-neoplastic therapy. Like the VAs, its mechanisms of action are based on preventing DNA synthesis and tubulin polymerization, thus blocking mitosis [129]. Treatment responsiveness to colchicine (as well as to other drugs) has been associated with adenosine triphosphate-binding cassette subfamily B member 1 (ABCB1) expression, whose genetic variations are associated with the pathogenesis of several dermatological diseases, including melanoma [135]. Despite some interesting new potential applications for anti-neoplastic therapy, its application in clinics is hampered by the occurrence of some side effects.

Berberine is an isoquinoline alkaloid that can be isolated from the bark and the roots of plants from *Berberis* genus and others. It was reported that berberine may exert anti-inflammatory, anti-neoplastic, anti-diabetic, anti-oxidant and other activities [136]. Recent studies reported a potent activity on melanoma cell viability, migration and invasion acting through inflammatory signaling pathways (e.g., NF-kB) [137] and increasing ROS production and apoptosis pathways [138].

Piperine is the alkaloid responsible for the pungency of black (*Piper nigrum* L.) and long pepper (*Piper longum* L.). Its growth inhibitory effects are mediated by cell cycle G1-arrest associated with DNA damage and induction of apoptosis [139] while other studies on murine melanoma cells showed that piperine treatment significantly reduced proinflammatory cytokines such as TNF-α, IL-1β and IL-6 [140]. The anti-inflammatory effects of piperine were also confirmed through in vivo studies when orally administered to a xenograft melanoma model [141]. Capsaicin is an alkaloid responsible for the pungency of chili peppers, which are plants belonging to the genus *Capsicum*. Recently it was demonstrated that this alkaloid is able to induce apoptosis and autophagy pathways in human melanoma cells [142]. Interestingly, other capsaicin effects on melanin biogenetic pathway in melanoma cells were reported [143,144], opening other interesting ways to explain the anti-inflammatory effects of this alkaloid.

Harmaline and harmalol are β-carboline alkaloids present in *Peganum harmala* L., a perennial herb from the Zygophyllaceae family with known anti-bacterial, anti-fungal and anti-viral activities [145]. These alkaloids were demonstrated to stimulate tyrosinase activity and melanin synthesis and to induce melanogenesis through the activation of p38 MAPK signaling [146].

Ipobscurine, an indole alkaloid from *Ipomoea obscura* (L.) Ker Gawl., was demonstrated to inhibit the proliferation, migration and invasion of murine melanoma cells and to reduce metastatic dissemination, as well as to exert in vivo anti-angiogenic and anti-inflammatory activities [147,148].

## 5. Phenolic Compounds

### 5.1. Flavonoids

**Figure 7:**
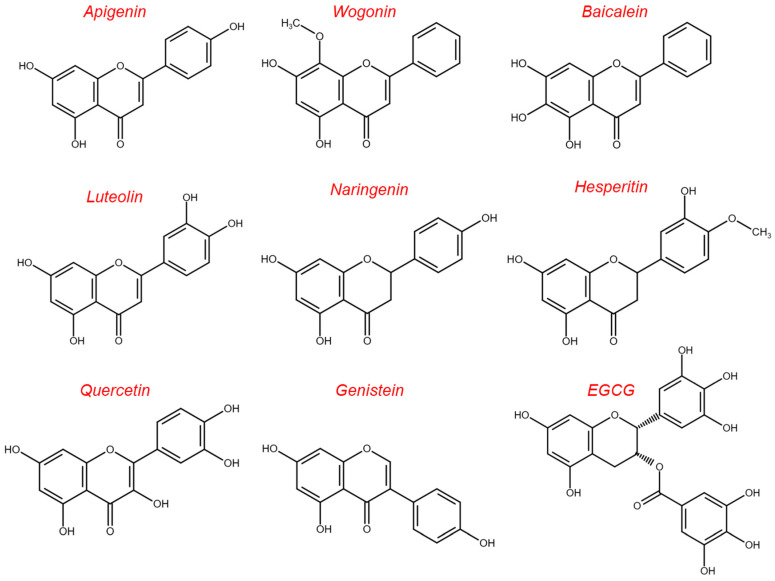
Chemical structure of principal flavonoids discussed in this section. EGCG, epigallocatechin gallate.

Flavonoids represent a class of secondary metabolites widely present in fruits and, in general, in foods and beverages of plant origins [149]. They are characterized by a common carbon skeleton (C6–C3–C6) in which the two aromatic rings A and B are connected by the oxygenated heterocyclic ring C (Figure 4). Flavonoids in general, and their aglycone forms in particular, exhibit a broad spectrum of bioactivities including anti-oxidant, anti-inflammatory and anti-cancer ones [150,151]. Their antioxidant effects are mediated by scavenging ROS, chelating metal ions, activating the anti-oxidant enzymes (e.g., superoxide dismutase (SOD), catalase (CAT)), and inhibiting oxidases (e.g., cyclooxygenase (COX) and lipoxygenase) [152]. Specifically COX-2 inhibition, as well as the ability to regulate nuclear factor-κB (NF-κB), activator protein 1 (AP-1), and mitogen-activated protein kinase (MAPK) represent the intracellular changes conferring anti-inflammatory activity to flavonoids [153]. In particular, these phytochemicals are able to reduce the levels of several pro-inflammatory mediators (e.g., TNF-α, IL-1β, IL-6, and IL-8) [153]. It is important to emphasize that the ability of flavonoids, and in general of dietary secondary metabolites, to modulate cellular and molecular inflammation-associated pathways and the capacity to inhibit both secretion and expression of pro-inflammatory cytokines is due to their direct interaction with immune cells [154,155]. However, this section mainly focuses on the direct effects of flavonoids on melanoma cells (Figure 7).

The anti-melanoma activity of flavones, mainly apigenin (4′,5,7-trihydroxyflavone), is well documented. Parsley, chamomile, celery and oregano represent the principal food sources of apigenin even in the dried form [156]. Apigenin, along with a significant pro-apoptotic capacity in melanoma cells, has been shown to exhibit inhibitory effects on IFN-γ-induced expression of PD-L1 through inhibition of STAT1 phosphorylation [157]. A recent study showed that the total extract from *Rhamnus alaternus* L., a wild Mediterranean plant of the Rhamnaceae family particularly rich in flavones content, exhibited in vivo anti-melanoma activity in the B16-F10 syngeneic murine model. Together with the reduction in the volume and weight of tumor, this plant extract increased the level of IL-2 in the bloodstream and reduced those of IL-6 during metastasis formation [158]. Wogonin (5,7-dihydroxy-8-methoxy flavone), a flavone found in *Scutellaria baicalensis* Georgi., potentiated TRAIL (TNF-α-related apoptosis-inducing ligand)-mediated apoptosis in SK-MEL-37 melanoma cells [159]. Wogonin up-regulated TRAIL-R2, resulting in the induction of cell death in melanoma [159]. Interestingly, a recent study demonstrated that baicalein, another flavone from *S. baicalensis*, inhibited melanoma growth in vivo by promoting the infiltration of tumor-associated macrophages (TAMs) and inducing the M1-like phenotype polarization [160]. Vegetables and fruits such as cereals, chicory, oregano, broccoli and carrots are rich in luteolin (3’,4’,5,7-tetrahydroxy flavone), a flavone that is able to activate antigen-presenting cells in B16-F10 in vivo models [161]. In this study, it was also demonstrated that luteolin plus antigen-vaccine activated CD8^+^ T cells improved the survival of the melanoma-bearing mice [161]. The immunomodulatory ability of luteolin has also been highlighted in melanoma cell lines through transcriptomic and bioinformatics approaches [162].

Quercetin (3,3′,4′,5,7-pentahydroxy flavone), a dietary flavonol present in onions, peas, apples and berries, is one of the most representative flavonoids in nature with a well-known anti-inflammatory activity [151]. In detail, quercetin inhibited melanoma cell proliferation and invasion through RIG-I (retinoic acid-inducible gene I)-induced upregulation of IFN-α and IFN-β expression, resulting in activation of the STAT1 signaling [163]. Moreover, the efficacy of quercetin–ferrum nanoparticles in enhancing the efficacy of photothermal therapy and in regulating (through reduction of PD-L1) the immunosuppressive microenvironment in primary melanoma has been recently demonstrated [164].

Naringenin (4′,5,7-trihydroxy flavanone) belongs to the group of flavanones and is found mainly in citrus fruits and tomatoes. Naringenin, in combination with asiatic acid (a triterpene from *Centella asiatica* [L.] Urb.), was shown to enhance NK cell-mediated innate immune response and suppress melanoma invasion via TGF-β/Smad signaling [165]. Similarly to what was previously reported for luteolin, another flavanone from citrus fruits, hesperidin (its aglycone form is called hesperetin), inhibited the progression of melanoma in tumor-bearing mice by activating CD8^+^ T cells and promoting IL-6 secretion by activating the PI3K-AKT pathway [166].

Isoflavones, including genistein and daidzein, are phytoestrogens, mainly present in members of the Fabaceae family (soy, lentil, and bean). They have attracted much attention following epidemiological studies, which have highlighted their protective effect against hormone-dependent tumors [167]. In addition, their anti-melanoma and immunomodulatory activity has also been reported. Genistein (4,7,4’-trihydroxy-isoflavone) hampers B16-F10 tumor growth by dose-dependently cytotoxic T-cell activity, IL-2-stimulated NK activity, and basal splenocyte proliferation [168]. Interestingly, another study clearly demonstrated that genistein affects the progression of oral, uveal and cutaneous melanoma by reducing the PGE2-induced IL-8 expression via the EP3 receptor [169].

Catechins, also known as flavan-3-ols, are present in a large variety of foods and beverages such as berries, kiwi, grape seeds, cocoa, red wine and green tea. Tea catechins, such as epigallocatechin gallate (EGCG) which is the most abundant and studied catechin of green tea (*C. sinensis* leaves), as well as epigallocatechin and epicatechin gallate have attracted much attention for their effective anti-oxidant activity [170]. Notably, EGCG potentiated the pro-apoptotic effects of both IFN-α2b in human melanoma cell lines A375, Hs-294T and G-361 [171], and of TRAIL in A375 cells [172], similar to what was previously reported for wogonin. It has also been reported that low doses of EGCG suppressed melanoma cell growth through inhibition of NF-kB with parallel reduction of IL-1β secretion from melanoma cells. In particular, Ellis and colleagues [173] clearly demonstrated that EGCG down-regulated the NLRP1 (NLR Family Pyrin Domain Containing 1) protein, a component of inflammasomes, resulting in a reduction of IL-1β and inactivation of NF-kB. Furthermore, in combination with the hypoglycemic agent metformin, a drug largely used for type 2 diabetes, the EGCG inhibited the NF-kB/STAT3 signaling pathways, resulting in reduced melanoma growth and metastatic potential, and decreased pro-inflammatory mediators such as IL-6, IL-10 and TNF-α [174]. The possibility to use EGCG in combination with DNA vaccination in order to potentiate tumor-specific T-cell immune responses has been also explored [175]. The results demonstrated that EGCG was able to increase specific CD8^+^ T cells generated by DNA vaccine in mice but, on the contrary, higher doses of EGCG exerted potential immunosuppressive effects [175]. It has been recently demonstrated that EGCG inhibits IFN-γ-induced PD-L1 and PD-L2 expression, and JAK-STAT signaling in melanoma cells, while in animal studies EGCG blocked PD-L1 expression, leading to the activation of CD8^+^ T cells [176].

Several studies have emphasized the anti-proliferative effects of anthocyanidins and their aglycone form, known as anthocyanins, on melanoma cells [177,178]. However, to our knowledge, there are no data supporting a direct immunomodulatory activity of these flavonoids on melanoma cells.

Another very interesting aspect of flavonoids is their potential use in chemoprevention, to reverse or prevent the initial stages of carcinogenesis or the progression of malignant cells. The chemopreventive effects of anthocyanins in skin cancer have been well-documented [179]. For example, the potential of topical application of pomegranate (*Punica granatum* L.) extract, which contains high levels of anthocyanins, such as delphinidin, cyanidin and pelargonidin, and ellagitannins, such as punicalagin, has been demonstrated. This extract prevented the TPA-induced skin tumorigenesis in CD1 mice through the inhibition of COX-2, MAPKs and NF-kB [180].

Furthermore, flavonoids have also been shown to possess photoprotective abilities [181,182]. UV radiation represents one of the riskiest environmental factors for skin, causing erythema, edema, photoaging, and finally skin cancers such as melanoma. UV radiation, in addition to the direct effect producing adducts between DNA bases, modifies several signaling pathways associated with oxidative stress and inflammation, thus impairing skin health by increasing the formation of ROS [183]. It has been demonstrated that liposomes containing glabridin, a isoflavone found in licorice (*Glycyrrhiza glabra* L.), counteract the UVB-induced skin damages by reducing TNF-α, IL-6 and IL-10 levels [183]. In addition, the inhibition of photocarginogenesis has been demonstrated for genistein [184], silymarin (a mixture of flavonolignans and flavonoid from *Silybum marianum* (L.) Gaertn) [185], EGCG [186] and others.

Aberrant gene expression is a hallmark of cancer. DNA methylation and histone modifications are well-known epigenetic mechanisms able to modulate gene expression in various ways. They are often dysregulated in various cellular processes as cell cycle, proliferation and apoptosis and this leads to cancer itself. In skin cancer as well as in all other types of cancer, these processes are dysregulated. In melanoma, DNA gene promoter hypermethylation with consequent transcriptional silencing has been observed for tumor suppressor genes as *PTEN*, *CDKN2A* and *RASSF1A* [187]. In addition to these gene promoter-specific changes in DNA methylation, a genome-wide DNA hypomethylation of chromosomal centromeric regions leading to genomic instability has been documented in different cancer types including melanoma. Indeed, DNA methyltransferases (DNMTs) have been found to be dysregulated in cancer. The ability of polyphenols to modulate immune system, through the activation and differentiation of immune cells, by epigenetic mechanisms has been well documented [6]. Interesting results were obtained using tea catechins of which EGCG has been shown to have chemopreventive and chemotherapeutic activities. EGCG suppresses DNA methylation by down-regulating DNMTs in SCC-13 and A431 skin cancer cell lines [188], whereas in another study [189] it reactivated the expression of specific tumor suppressor genes such as p21 and p16INK4a by decreasing their promoter methylation and by increasing histone acetylation. EGCG has also been shown to influence specific histone methylation (lysine 9 of H3), leading to upregulation of tumor suppressor genes such as p21 and p27 in human skin cancer cell lines [190]. Polyphenols from green tea act also on histone deacetylases (HDACs) by decreasing their expression and hence enhancing the expression of some tumor suppressor genes including p16, p53 and p21 in melanoma cells [191].

### 5.2. Phenolic Acids

The beneficial effects of phenolic acids, including anti-oxidant, anti-inflammatory and immunostimulating effects, are largely investigated in melanoma and were recently reviewed [192]. Their activity is often limited by the low bioavailability, which reduces the actual concentration achieved within the tissues. An effective strategy currently tested is their conjugation with phospholipids [193]. The immunostimulating effects of phenolic acids such as caffeic acid, *p*-coumaric acid, and ferulic acid has been found to be mediated by the release of NO, ROS, and cytokines including TNF-α, IL-1β, IL-6 [194], and IL-4 [195]. Cytotoxic effects as well as immune stimulation promoting T cell proliferation were also recently found in plant extracts from Mauritius island, containing flavonoids and phenolic acids with anti-melanoma effects observed in vitro in B16-F10 mouse melanoma cells [196]. Further, the phenolic compound named gallic acid synthesized conjugated with NLG8189 has shown a clear immunomodulatory action in melanoma setup, regulating the CD4^+^/CD8^+^ ratio and the number of Treg cells, as well as significantly inhibiting melanoma growth both in vitro and in vivo [197]. Compounds containing phenolic acids such as gallic acid and ethyl gallate, extracted form *Caesalpinia spinosa* (Molina) Kuntze, induce a cooperative autophagy-based immunomodulatory mechanism known as immunogenic cell death, observed in melanoma murine models [198], and also showed a direct anti-proliferation activity in melanoma- as well as non-melanoma cancer cells [199]. The immunomodulatory action of caffeic acid and caffeic acid phenethyl ester leads to anti-melanoma effects via the inhibition of PI3K/AKT/XIAP pathway in both mouse and human melanoma cells by suppressing the activating phosphorylation of phosphoinositide 3-kinase and phosphoinositide-dependent kinase-1 and AKT [200]. It also reduces expression of XIAP, survivin and BCL-2, and increases XIAP nuclear translocation, resulting in apoptosis promotion [200]. Caffeic acid also inhibits IL-10 expression, thus protecting against UVB-induced immune suppression [201]. Interestingly, several phenolic acids are known to affect melanogenesis and thyrosinase activity; this field has been recently reviewed [202] and is under continuous investigation in melanoma in vitro systems [203,204,205]. Salicylic acid, one of the main ginseng root phenolic acid components, reduces melanin levels in mouse B16-F10 melanoma cells by inhibiting tyrosinase and tyrosinase-related proteins, as well as their master transcriptional regulator, microphthalmia-associated transcription factor (MITF) and other upstream signal regulators such as adenylyl cyclase and protein kinase A [206]. Other pumpkin-derived polyphenolic compounds such as coumaric acid show a clear anti-oxidant and anti-tyrosinase activity in mouse B16-F10 melanoma cells [207] and a relevant anti-proliferative effect down-regulating cycle-related proteins, up-regulating Apaf1 and BAX and down-regulating BCL-2, ultimately inducing apoptosis in both human and mouse melanoma cells [208].

### 5.3. Stilbenes

Resveratrol (trans-3,5,4′-trihydroxystilbene) belongs to the polyhydroxystilbene subclass of plant polyphenols and was first isolated in 1939 from the roots of *Veratrum grandiflorum* O. Loes. It is well-known for its anti-oxidant and anti-inflammatory effects especially on the cardiovascular system [209]. Resveratrol strongly modulates immune responses both in vitro, in human cells and in vivo, in mouse models (reviewed in [210]). Some of these studied were performed in melanoma cells or murine melanoma xenografts [211]. Resveratrol functions in immune system are partially mediated by enhancing the activity of the sirtuin-1 (SIRT1) deacetylases, known to maintain periphery T cell tolerance. Activation of SIRT1 by resveratrol leads to decreased NF-kB-induced expression of inflammatory factors such as TNF-α, IL-1β, IL-6, MMP-1, MMP-3, and COX-2 [212]. However, resveratrol also acts on p300 expression and promotes inhibitor protein-kBα (IkBα) degradation, probably independently from SIRT1. Resveratrol modulates both innate and adaptive immunity. It induces an anti-inflammatory profile in macrophages, and inhibits the nod-like receptor family, pyrin domain containing 3 (NLRP3)-inflammasome activation [213]. On T cell activation, resveratrol exerts an inhibitory function in autoimmune diseases, whereas in tumor models it reduces the suppressive function of Tregs, inhibiting tumor growth. Interestingly, resveratrol induces a significant enhancement at low concentrations and suppression at high concentrations of both cytotoxic T lymphocytes and NK cell cytotoxic activity [214]. Dose-dependent diverse effects of resveratrol were also seen in the human THP-1 monocyte/macrophage model. Here, low doses of resveratrol inhibit cell proliferation with induction of a S phase arrest; at higher concentrations, resveratrol induces cell apoptosis and causes G0/G1 phase arrest [215]. Additional studies on immune response to tumors generally showed that high doses of resveratrol are cytotoxic for both cancer and beneficial effector immune cells, whereas low- and non-cytotoxic doses of resveratrol inhibit the generation and function of tumor-evoked regulatory B-cells (tBregs), including their expression of TGF-β and their ability to convert T lymphocytes into Foxp3+ Tregs, blocking tumor metastatic spreading [216]. This effect on tBregs is achieved by inactivating STAT3 phosphorylation and acetylation [216]. In melanoma, treatment with resveratrol significantly reduces IL-17 secretion and suppresses Th17 activity [217]. Moreover, in T lymphocytes, resveratrol modulates PD-L1 glycosylation, promoting the endoplasmic reticulum retention of the abnormally glycosylated PD-L1 and enhancing anti-tumor T cell immunity [218].

Even if resveratrol is the most well-known and studied compound of the stilbene class, other compounds have been tested for potential clinical use. Among these, Gnetin-C, a dimer of resveratrol, has been isolated from Gnetaceae and Polygonaceae plant families. Like resveratrol, Gnetin-C has anti-oxidant, anti-microbial and anti-inflammatory properties. Healthy volunteers treated with Gnetin-C showed increased levels of circulating NK cells with higher cytotoxic potential and a decrease in the number of circulating neutrophils [219]. Resveratrol has also been investigated in combination with curcumin and synergistically activates host immunity and inhibits cancer development with limited side effects [220].

### 5.4. Other Phenolic Compounds

At least 1300 different coumarins have been identified, as recently pointed out [221]. Coumarin-related molecules show, among others, direct anti-viral activity [221], anti-inflammatory and anti-melanoma activity [222]. While the mechanism of action is still under investigation, coumarin’s ability to reduce neutrophil infiltration and inflammatory reaction has been demonstrated in a rheumatoid arthritis model [223] and coumarin metabolites induce IL-12 expression in vitro in murine macrophages, and show anti-tumor effect using an in vivo murine sarcoma model. Coumarin is known to enhance HLA-DR and HLA-DQ antigen expression in peripheral blood mononuclear cells, indicating an activation state of these cells [224]. Coumarin derivatives show both immunosuppressive [225] and immunostimulatory effects [226], the latter being mediated by an increase of CD4^+^ and CD8^+^ T cells. Some coumarin derivatives show cytotoxic and pro-apoptotic effects in human melanoma cells [227,228] and opposite effects on melanogenesis, either inhibitory, achieved by direct inhibition of tyrosinase activity and of melanogenesis-related genes expression [229], or stimulatory, with a dose dependent proliferation enhancement of B16 murine melanoma cells.

**Figure 8:**
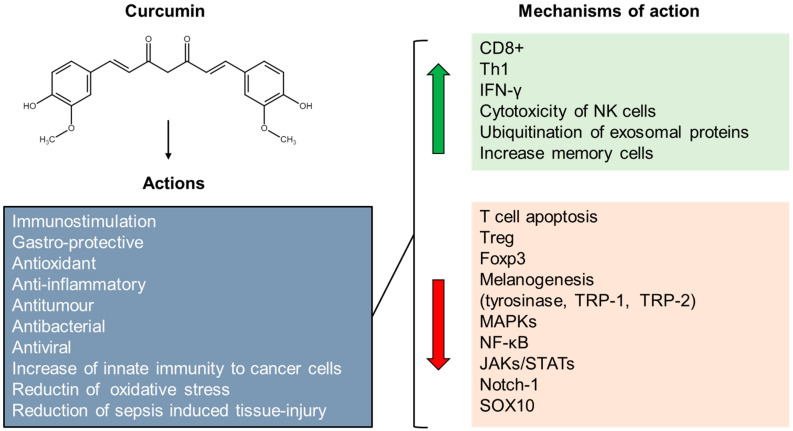
Mechanisms of action of curcumin.

Several pieces of evidence show that curcumin and curcumin-related molecules exert a significant anti-melanoma effect in vitro [230,231,232], suggesting them as promising candidates for future melanoma treatment [233]. As recently published [234], curcumin treatment leads to inhibition of SOX10 and Notch1 expression in human melanoma cells, as well as to increased expression of miR-222-3p, which is reported to modulate immune cell function [235]. Further, as recently reviewed [236], curcumin has a strong impact on immune system functions by increasing the number of CD8^+^ Tcells, stimulating the Treg switch to Th1 cells, reducing the number of Treg cells, inhibiting Foxp3 and inducing IFN-γ expression. Like phenolic acids, curcumin also inhibits melanogenesis [237] by inhibiting tyrosinase activity and expression of melanogenesis-related molecules such as TRP-1 and TRP-2. Therefore, curcumin may share at least part of the mechanism of action with some phenolic acids (Figure 8). A direct action of curcumin on gut microbiota has also been reported [238], indicating an additional way it may act to control immune response.

**Figure 9:**
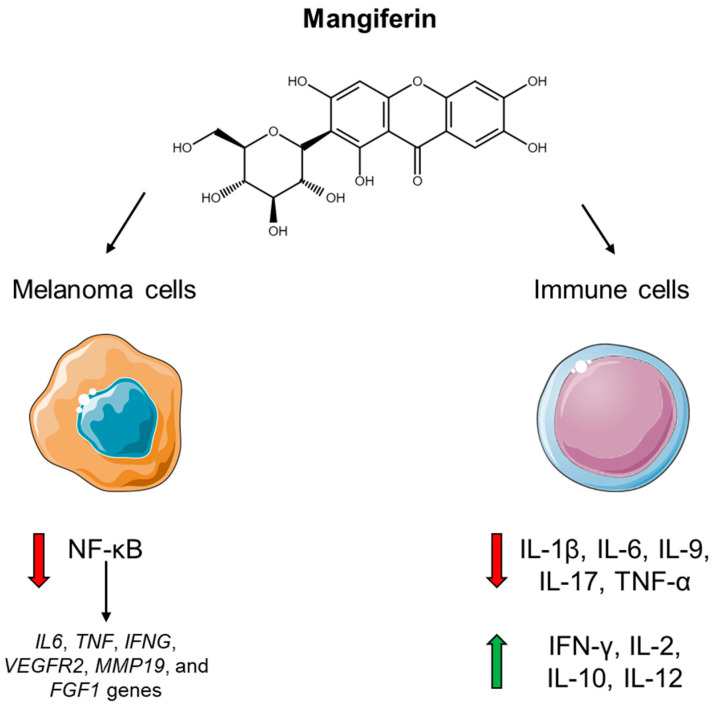
Mechanisms of action on melanoma and immune cells. Parts of the figure are drawn using pictures from Servier Medical Art (https://smart.servier.com, (accessed on 20 January 2023)).

Gambogic acid is a polyprenylated xanthone, extracted from the resin of the traditional Chinese medical plant *Garciania hanburyi* Hook.f., that exhibits anti-tumor activity. Gambogic acid significantly inhibits the migratory, invasive and adhesive properties of melanoma cells and in vitro angiogenesis. This in vitro anti-metastasis effect is mediated by inhibition of the PI3K/Akt and ERK signaling pathways that result in the suppression of the EMT and angiogenesis [239]. Mangiferin is a C-glucosyl xanthone and exerts many beneficial biological activities such as anti-oxidative and anti-inflammatory ones. Mangiferin is present in diverse plants but mostly in the *Mangifera indica* L. (mango tree) of the Anacardiaceae family. Its free radical-scavenging activity is attributed to the xanthonoid structure and is therefore common to other xanthones. Besides being cytotoxic for tumor cells in vitro, consumption of mangiferin could have a prophylactic role against acute and chronic inflammation and cancer. Mangiferin, in fact, shows cytoprotective and anti-genotoxic properties [240]. The effect of mangiferin on melanoma metastasis and tumor growth was assessed in vivo in a mouse model where mangiferin inhibited both spontaneous metastasis and tumor growth [241]. Mangiferin interferes with inflammation through inhibition of several NF-κB target genes. In particular, gene expression profiling of B16-F10 melanoma cells reveals that mangiferin selectively inhibits expression, among others, of *IL6*, *TNF*, *IFNG*, *VEGFR2*, *MMP19* and *FGF1* genes with consequent deregulation of angiogenesis, cell viability and metastatic processes [242]. Mangiferin modulates immune responses through multiple mechanisms: (1) reducing eosinophils and total inflammatory cells infiltration in the tissue; (2) decreasing prostaglandin presence in the patient serum; (3) down-regulating an amount of circulating Th2-related cytokines/chemokines such as IL-1β, IL-6, IL-9, IL-17, TNF-α; (4) increasing serum levels of Th1-related chemokines such as IFN-γ, IL-2, IL-10, IL-12; (5) exerting an immunoprotective role via inhibition of oxidative stress in lymphocytes, neutrophils and macrophages [243] (Figure 9).

Shikonin is a naphthoquinone from the root of *Lithospermum erythrorhizon* Sieb. et Zucc. (Boraginaceae), and exhibited anti-angiogenic properties in a B16 melanoma model [244]. In addition, shikonin induces high expression level of RANTES, and shows promising immune-modulatory activity as an adjuvant in a human gp100-transfected B16 mouse tumor model [245].

A multitude of studies regarding the effects of phytonutrients on melanoma focused on Aloe plants containing an important amount of natural products. Among them was studied its natural hydroxyanthraquinone Aloe-emodin (AE) for its possible anti-melanoma properties [246,247]. It is a hydrophobic compound, which presents itself as a crystal. Its pharmacological effects are closely linked to its structure, in particular the anthraquinone ring and is the pharmacophore responsible for its important biological properties, including anti-tumor, anti-oxidant, anti-microbial, anti-bacterial and anti-fungal [247]. AE has been shown in many studies to reduce the viability and proliferation of various cancer cell species by inducing apoptosis, but also by inhibiting adhesion and migration [248]. Due to its planar nature, it manages to bind, non-covalently, to DNA and to the enzyme topoisomerase II, inhibiting the DNA locking reaction after double-strand cleavage. One of AE’s main problems is that it is poorly soluble in water, which greatly complicates its use in various therapies. What has been considered in recent years is to introduce it into nanocarriers to overcome this problem, but also to have a longer release time to improve its effect [249,250]. Aloe-emodin reduced the growth of mouse B16 and human A375 melanoma cells by two different signaling pathways through ROS production via activation of ERK1/2 as demonstrated by Radovic et al. [251]. The anti-melanoma effects of AE included time-dependent anti-proliferation, inhibition of polyamine metabolism, and induction of differentiation (through the induction of tissue transglutaminase activity) [252]. Anti-metastatic activities of AE induced deep alterations of human melanoma cells mechanisms of aggregation, migration, adhesion and invasion of murine melanoma cells [252]. AE also induced cell differentiation on human melanoma cells [253]. Moreover, Tabolacci and colleagues [253] demonstrated that AE increased the expression of inflammation-associated factors such as interleukin IL-2, IL-12, GM-CSF and IFN-γ, and showed an immunomodulatory property against different human melanoma cells lines as A375 and SK-MEL-28 cells. Lin et al. [254] determined that AE reduced the growth of human malignant melanoma A375.S2 cells by inhibiting the enzyme activity of N-acetyltransferase. As known, understanding these immune cells, and their specific antigens, could allow modulating the immune system. In melanoma’s TME, numerous interactions implicated in the pro-tumor or anti-tumor response are established between many immune cell subsets. The beneficial immunomodulatory properties of AE on immune cells have been studied in various models [255]. Additionally, all the effects observed during treatment with this natural compound have been described both in vitro and in vivo evaluations of the immune system activities mediated by murine and human lymphoid cells. In particular, AE increased the levels of IL-1β and TNF-α [256,257].

Lignin is a highly heterogeneous polymeric natural product abundantly found in plants. *Lentinula edodes* (Berk.) Pegler, is a common edible mushroom in Japan and China. Cultured *L. edodes* secrete a lignin-degrading peroxidase that produces low molecular weight lignin hydrolysate. These mushroom extracts inhibit melanoma growth in tumor-bearing mice by restoring immune responses of class I-restricted and melanoma-reactive CD8+ T cells and by reducing Treg immunosuppression [258]. A lignin–carbohydrate complex was isolated from *Pimpinella anisum* L., the aromatic herb anise, and possesses similar anti-viral and immunostimulating effects, inducing iNOS, IL-1β and IL-10 mRNA expression in macrophages [259]. Analogous lignin–carbohydrate complexes have been isolated from other plants and have been mainly characterized for their anti-viral activity, but an immunomodulating role has been assessed as well [260].

Lignans constitute a large group of phenylpropanoid metabolites found in diverse plants [261]. Lignans can be divided into different structural types and the most common compounds are the dibenzyl butyrolactones (arctigenin and savinin), furofurans (sesamin) and dibenzyl butanes. Arctigenin has been isolated from the seeds of *Arctium lappa* L., and exhibits therapeutic effects on some autoimmune diseases, especially immunological hepatitis. It inhibits T cell proliferation, Th17 differentiation, macrophage activation and the release of pro-inflammatory cytokines [262]. Less studied than arctigenin, the *Pterocarpus santalinus* (L.)-derived lignan savinin also displays anti-inflammatory properties, regulating both TNF-α production and T cell proliferation [263]. Schisandrin is a bioactive dibenzocyclooctadiene lignan present in the fruit of *Schisandra chinensis* (Turcz.) Baill. which is used, together with Schisandrin B and C, in traditional Chinese medicine as an anti-tumor, anti-inflammatory and immunomodulatory molecule. Schisandrin B and C reduce the production of IL-1β, TNF-α, IL-6, IL-10, and IL-12 by stimulated macrophages, and alter the cellular redox state in both macrophages and dendritic cells [264]. Moreover, Schisandrin B promotes Treg expansion and modulates Th17 cell differentiation [265]. Similarly, anwulignan, a monomer of *Schisandra sphenanthera* Rehd. et Wits lignans, has anti-oxidant and immunomodulatory effects. Anwulignan counteracts immunosenescence by increasing of serum levels of IL-2, IL-4, and IFN-γ, decreasing levels of TNF-α and IL-6, as well as by activating the Nrf2/ARE pathway and diminishing apoptosis in the spleen [266]. Sesamin is a lipid-soluble furanofuran-type lignan from *Sesamum indicum* L. seeds and sesame oil recognized for its immunomodulatory and anti-inflammatory properties. Oral administration of sesamin leads to significant suppression of IL-6 and TNF-α expression in mice [267]. Sesamin also exerts anti-tumor effects via enhancing NK cell cytotoxic activity [268].

## 6. Terpenes and Terpenoids

Terpenoids, also referred to as terpenes, have been comprehensively studied and reported to play key roles in humans health interchangeably. They possess a wide range of biological activities including anti-cancer, anti-parasitic, anti-microbial, anti-allergenic, anti-spasmodic, anti-hyperglycemic, anti-inflammatory and immunomodulatory properties [269]. The current literature involving in vitro and in vivo studies indicates the potential of various terpenes as suitable immunomodulators for the alternative/adjuvant treatment of cancer, including melanoma. They can activate our immune system by enhancing both humoral and cell-mediated immune responses. In particular, they can activate anti-cancer immunity, which may be involved in anti-cancer and anti-metastatic potential [270,271].

The triterpene Glycyrrhizic acid (18β-GL or GL), the main bioactive ingredient of licorice *G. glabra* L. (Fabaceae), is evidenced to have several immunomodulatory activities [272,273]. In particular, Raphael and Kuttan demonstrated that administration of glycyrrhizic acid clearly enhanced the level of interleukin IL-2, the antibody-dependent cell mediated cytotoxicity (ADCC) and antibody-dependent complement-mediated cytotoxicity (ACC) in B16-F10 metastatic melanoma-bearing mice [274]. It also stimulated macrophage-derived NO production and was able to up-regulate inducible nitric oxide synthase (iNOS) expression through NF-kB transactivation in murine macrophages [275]. GL and glycyrrhetinic acid could induce IFN-γ activity and augment NK cell activity [276].

Ursolic acid (UA) is a pentacyclic triterpenoid found in various fruits, vegetables and medicinal herbs and has a broad range of biological effects [277]. In vitro, UA could induce apoptosis in B16-F10 melanoma cells by p53-induced caspase-3 activation and inhibition of NF-kB-mediated activation of bcl-2 [278]. UA could significantly inhibit the production of TNF-α, IL-1β, IL-6 and GM-CSF gene expression and production by B16F-10 melanoma cell in culture in a dose-dependent manner. In vivo, UA was found to stimulate immune system by activating the cell-mediated immune responses in B16-F10 melanoma-bearing mice. Intraperitoneal administration of UA was found to enhance ACC and antibody-dependent cell-mediated cytotoxicity ADCC and produce increased NK cell activity in metastatic tumor-bearing animals. Moreover, the reduction of GM-CSF and IL-6 levels was observed in vivo after treatment with UA. On the other hand, the level of IL-2 was enhanced by the treatment with UA compared with untreated tumor-bearing control animals [274].

Nomilin is a triterpenoid present in common edible citrus fruits with putative anticancer properties. In B16-F10 melanoma cells, treatment of nomilin exhibited a down-regulated Bcl-2, cyclin D1 expression, and up-regulated p53, Bax, caspase-9, caspase-3, p21, and p27 gene expression. The production and expression of pro-inflammatory cytokines such as IL-1β, TNF-α, IL-6, and GM-CSF were found to be down-regulated in nomilin-treated cells. Nomilin also could inhibit the activation and nuclear translocation of anti-apoptotic transcription factors such as NF-kB, CREB, and ATF-2 in B16-F10 cells [279].

Andrographolide is a labdane diterpenoid isolated from the leaves and roots of *Andrographis paniculata* (Burm.f.) Wall. and has shown anti-cancer and anti-inflammatory potential. Intraperitoneal administration of andrographolide significantly inhibited the B16-F10 melanoma cell line-induced capillary formation in C57BL/6 mice and reduced the levels of pro-inflammatory cytokines, such as IL-1β, IL-6, TNF-α and GM-CSF, and serum NO level [280].

Limonene is a naturally occurring monoterpene that serves as a precursor of other oxygenated monocyclic monoterpenes such as carveol, carvone, menthol, perillyl alcohol (POH) and perillaldehyde. D-Limonene has well-established chemopreventive activity against many cancer types [281]. Kuttan et al. demonstrated that limonene reduced metastatic tumor nodule formation in a murine melanoma model. D-Limonene also was found to activate the immune system and increase the total antibody production and the number of antibody-producing cells in spleen and bone marrow [282].

Vernolide-A (C21H28O7) is a sesquiterpene lactone (SL) present in the plant *Vernonia cinerea* L. (Asteraceae). Pratheeshkumar and Kuttan investigated the effect of vernolide-A on the induction of apoptosis as well as its regulatory effect on the activation of transcription factors in B16-F10 melanoma cells. They found that Vernolide-A treatment, induced DNA fragmentation and up-regulated pro-apoptotic genes p53, Bax, caspase-9, and caspase-3. The study also reveals that vernolide-A treatment could alter the production and expression of pro-inflammatory cytokines and inhibit the activation and nuclear translocation of p65, p50 and c-Rel subunits of NF-kB, and other transcription factors such as c-fos, activated transcription factor-2, and cyclic adenosine monophosphate response element-binding protein in B16-F10 melanoma cells [283].

The last example we report in this paragraph is the Terpinen-4-ol the main monoterpene component of Tea tree oil (TTO; obtained from the leaves of *Melaleuca alternifolia* Cheel). TTO is responsible of its efficacy on melanoma cells. Several studies demonstrated that terpinel-4-ol can affect melanoma cell proliferation/viability, overcoming the resistance to caspase-dependent apoptosis, and interfere with the migration and invasion processes of melanoma M14 cells impairing the ERM (Ezrin, Radixin and Moesin)-mediated MAPK signaling pathway [284,285,286]. Another study pointed out to cytoskeleton as a further target of terpinen-4-ol and could account for the inhibition of proliferation and invasion of skin transformed M14 cells [287]. Like many other terpenes, terpinen has also shown immunomodulating properties. In particular, it was found that it can significantly reduced the production of pro-inflammatory cytokines, TNF-α, IL-1β, IL-6, IL-8 and IL-10 of monocytes using in vitro and in vivo assays [288,289].

## 7. Sulfur-Containing Compounds

Isothiocyanates (ITCs) are small molecules derived from glucosinolate precursors, naturally present into cruciferous vegetables. Due to their ability to reduce carcinogens activation and to increase their detoxification, they display anti-tumor and anti-inflammatory activities, mainly by affecting oxidative stress, MAPK signaling, deubiquitinating enzymes, cell cycle progression and apoptotic pathways. These effects were reported in several cancer cell types including human malignant melanoma cells [290,291,292]. Sulforaphane is probably one of the most studied ITCs whose potent activities as anti-cancer and epigenetic modulators were recently reviewed [293]. Further, the pro-apoptotic activity of sulforaphane on highly metastatic melanoma cells was demonstrated and associated with a significant caspases activation and down-regulation of pro-inflammatory cytokines such as TNF-α, IL-6, IL-1β, IL-12p40, and GM-CSF in murine melanoma cells [294]. Interestingly, a potent effect of sulforaphane in combination with quercetin on melanoma progression through MMP-9 down-regulation was reported in a mouse model [295]. On the other hand, recently the effects of sulforaphane to reduce melanoma cell proliferation and to increase tyrosinase production was reported [296].

As previously mentioned, epigenetic alterations play a key role in melanoma progression [297]. In addition to DNA methylation, histone modifications including mainly acetylation, methylation and phosphorylation also play a role in melanoma proliferation and altered expression of histone modifying enzymes such as HDACs has been reported. In general, HDACs remove the acetyl groups from the tails of histones leading to transcriptional repression. Other proteins such as Methyl-Binding Proteins (MBPs) bind to methylated DNA, causing gene silencing by recruiting other repressor complexes which often include HDACs [187]. Many studies have demonstrated that several phytochemicals are effective in preventing and reducing melanoma progression by acting on epigenetic mechanisms. Sulforaphane has been shown to modulate the activity of HDAC and of DNMT in many cancer types, including skin cancer. Saha et al. [190] demonstrated that polycomb proteins, which are considered negative epigenetic regulators, are upregulated in melanoma tumors, and contribute to transcription inactivation of cell cycle regulators by interacting with the PRC1/PRC2 histone methylation complexes. Another study [298] on mouse skin epidermal cells demonstrated that treatment with sulphoraphene significantly reduced their 12-O-tetradecanoylphorbol-13-acetate (TPA)-induced malignant transformation by enhancing the expression of the anti-cancer Nrf2 transcription factor. Nrf2 promoter DNA methylation was significantly reduced and the protein activity and expression of HDACs and the protein expression of DNMTs were also significantly reduced upon sulphoraphene treatment. Similarly, anthocyanin that is abundant in fruits and vegetables has been recently shown to activate Nrf2 by reducing its promoter methylation through protein reduction of specific DNMTs and HDACs [299]. Upregulation of Nrf2 by anthocyanin inhibited the neoplastic transformation of mouse skin JB6 P+ cells. Another study [300] demonstrated that HaCaT keratinocytes displayed down-regulation of HDAC protein levels following sulphorane treatment.

Allicin has been reported to stimulate the immune system and to exert anti-melanoma activity in a mouse model [301]; it also sensitizes human melanoma cells to the anti-proliferation effect of all-trans retinoic acid (ATRA) [302]. Along with other garlic components such as allyl sulfides, it inhibits melanoma growth as well as other cancers, with a mechanism including ROS increase, endoplasmic reticulum stress, apoptosis [303,304], and mitochondrial Ca^2+^ overload, dependent on Ca^2+^ channels. Interestingly, the anti-melanoma activity of diallyl trisulfide (DATS) is lost in the presence of Ca^2+^ channels blockers [305]. Garlic-derived compounds such as diallyl trisulfide and ajoene promote immune responses such as macrophage phagocytosis and activity of NK cells in leukemia mice [306] and other in vitro and in vivo models [307].

## 8. Discussion and Conclusions

An important issue deserving to be taken into account comes from the consideration that most of the studies cited within the present review are focused on the immunomodulating effects of phytochemicals on human or murine melanoma cells. Only a few studies are based on in vivo models of melanoma, mainly describing the effects of phytochemicals on the syngeneic murine model (i.e., B16-F10 murine melanoma cells in C57/BL6 mice), while other models based on human melanoma cells injected onto nude mice cannot be considered useful tools for investigations on immune-modulation [308]. On the other hand, the epidemiological/nutritional approaches on large population studies may represent a valid and potent strategy to investigate phytochemical effects on human cancers, including melanoma, with the limitation that in most cases the object of these studies are not the single natural compounds, but phytocomplexes, extracts or enriched foods which usually contain a mixture of many compounds. The potentiality of some phytocomplexes as anti-microbial or anti-hyperthensive agents was reported underlying the role of the active constituents [309,310]. On the other hand, the presence of many active compounds makes it very difficult to calculate the effects of each active molecule, since the extracts, foods and beverages may contain many phytochemicals differently combined and active, whose bioavailability may be also modified by the others. For instance, in the case of polyphenols’ bioavailability, as well as for other natural compounds, it was reported that is depends on many factors such as the interaction with the herbal and the food matrix, the chemical/physical characteristics of each compound, the stability under enzymatic, digestive (and also cooking) processes and their metabolic transformation by liver and intestinal microbiota and enzymes [311,312].

Several lines of evidence support that phytochemicals are essential for stimulating immune cell functions (phagocytosis, lymphocytes proliferation and differentiation, inhibition of pro-inflammatory cytokines) involved both in both innate and acquired immune response [313,314,315]. Different plant-derived bioactive compounds show potent immunomodulatory activities through diverse machanisms, including MAPK, PI3K/Akt, NF-κB and Wnt signaling pathways [314]. From this review, it is evident that phytochemicals or herbal extracts, although different pharmacological activities, represent a promising immunomodulators also in TME. Indeed, immunomodulatory interactions between immune cells, tumor cells, stromal cells, soluble factors, and extracellular matrix play a pivotal role in maintaining a pro-inflammatory microenvironment that regulates tumor progression [316]. From this point of view, the possibility of modulating and/or contributing to cytokines/chemokines secretion by melanoma cells is of great importance. Table 2 summarizes the cytokines, chemokynes and growth factors modulated by phytochemicals.

Another fascinating issue emerging in this review is the activity of phytochemicals on melanogenesis that may be proposed to potentially counteract the immunosuppression in melanoma. An exhaustive review of the literature regarding the phytochemicals active on melanogenesis is out of the scope of this review, and it has to be underlined that a large body of literature on natural compounds potentially useful to treat the pigmentation disorders is available [317].

The large number of phytochemicals known, whose only those more frequently stud-ied and characterized have been here reviewed, makes it difficult to draw any general and conclusive statement. Nevertheless, a couple of interesting considerations may be underlined. On one hand, the interest of researchers and oncologists on the potential use of phytochemicals as new promising immunomodulating agents against melanoma in the last years is rapidly growing. This allows us to hypothesize that natural compounds may become even more frequently new useful weapons against this type of skin cancer possibly also for chemoprevention purposes. On the other hand, among several known mechanisms of actions reported for the mentioned phytochemicals, the ability of several of them to specifically act on melanin biogenetic pathways is emerging as highly interesting and promising.

## Figures and Tables

**Table 1:** Principal plant secondary metabolite extraction methods.

Secondary Metabolites	Extraction Method	References
Alkaloids	Extraction using a “acid–base shakeout” (only for basic alkaloids)	[101]
	Extraction using alcoholic solvents (methanol or ethanol) or apolar solvents (e.g., chloroform) at room temperature or by refluxing, with the main drawbacks to extract also not alkaloid compounds and to use harmful solvents.	
	Extraction by supercritical CO_2_ (SF-CO_2_)	[102]
	Extraction by ionic liquids	[103]
	Extraction by natural deep eutectic solvents	[104]
	Extraction by microwave-assisted extractions	[105]
Phenolic compounds	Conventional methods, i.e., solid-liquid extractions, can be used. The recovery of phenolics from plant matrices is strictly influenced by solvent, extraction time and temperature, all factors which may affect extraction efficiency due the low stability at high temperature and in alkaline medium where phenols easily oxidize	[106]
	Alternative methods requiring ultrasounds, microwaves, supercritical fluids, pressurized liquids, or enzymes can be applied	[84]
Terpenes	Considering their apolar character, terpenes are traditionally extracted by apolar solvents, such as chloroform and n-hexane.	
	Extraction by SF-CO_2_	[107,108]
	Volatiles compounds can be also extracted by hydro distillation or steam distillation	[109]
Glucosinolates	The enzymatic degration of glucosinolates must be taken into consideration during the extraction, where myrosinase denaturation is a crucial step. Therefore, the most common procedure to isolate glucosinolates from plant consists of extraction in 70% methanol for 10 min at 75 °C or boiling water in order to denaturate the enzyme	[110]
Thiosulfinates	Allicin extraction from garlic can be carried out by solvent extraction, which requires long time extraction. Alternatively, SF-CO_2_, Ultrasonic-Assisted Extraction (UAE) or Pressurized Liquid Extraction (PLE) can be used although they requires two separated processes, the enzymatic and extraction ones. Therefore, subcritical water extraction (SWE) is a good option in order to have in a single-step the enzymatic and extraction process in a close system	[111]

**Table 2:** Immunomodulatory effects of plant secondary metabolites on in vitro and in vivo melanoma models.

Inflammatory Mediator	Secondary Metabolites	References
GM-CSF	Ipobscurine	[148]
	Aloe-emodin	[253]
	Glycyrrhizic acid	[274]
	Ursolic acid	[274]
	Nomilin	[279]
	Andrographolide	[280]
	Vernolide-A	[283]
	Sulforaphane	[294]
IFN-α	Quercetin	[163]
IFN-β	Quercetin	[163]
IFN-γ	Aloe-emodin	[253]
IL-1β	Theophylline	[126]
	Piperine	[140]
	Ipobscurine	[148]
	EGCG	[172,173]
	Nomilin	[279]
	Andrographolide	[280]
	Vernolide-A	[283]
	Sulforaphane	[294]
IL-2	Ipobscurine	[148]
	Aloe-emodin	[253]
	Glycyrrhizic acid	[274]
	Ursolic acid	[274]
IL-6	Piperine	[140]
	Ipobscurine	[148]
	Naringenin	[166]
	Glabridin	[183]
	Glycyrrhizic acid	[274]
	Ursolic acid	[274]
	Nomilin	[279]
	Andrographolide	[280]
	Vernolide-A	[283]
	Sulforaphane	[294]
IL-10	Glabridin	[183]
	Caffeic acid	[201]
IL-12	Theophylline	[126]
	Aloe-emodin	[253]
	Sulforaphane	[294]
IL-17	Resveratrol	[217]
IP-10	Caffeine	[125]
	Theophylline	[126]
MCP-1	Theophylline	[126]
MIP-1α	Caffeine	[125]
	Theophylline	[126]
MIP-1β	Caffeine	[125]
	Theophylline	[126]
RANTES	Caffeine	[125]
	Theophylline	[126]
	Shikonin	[243]
TNF-α	Methylxanthines	[117]
	Piperine	[140]
	Ipobscurine	[148]
	Glabridin	[183]
	Shikonin	[243]
	Nomilin	[279]
	Andrographolide	[280]
	Vernolide-A	[283]
	Sulforaphane	[294]
VEGF	Theophylline	[126]
	Ipobscurine	[148]

## Data Availability

Not applicable.
